# Deamidation-related blood biomarkers show promise for early diagnostics of neurodegeneration

**DOI:** 10.1186/s40364-022-00435-8

**Published:** 2022-12-27

**Authors:** Jijing Wang, Ya-Ru Zhang, Xue-Ning Shen, Jinming Han, Mei Cui, Lan Tan, Qiang Dong, Roman A. Zubarev, Jin-Tai Yu

**Affiliations:** 1 Department of Medical Biochemistry and Biophysics, Karolinska Institutet, Stockholm, Sweden; 2 Department of Neurology and Institute of Neurology, Huashan Hospital, State Key Laboratory of Medical Neurobiology and MOE Frontiers Center for Brain Science, Shanghai Medical College, Fudan University, Shanghai, China; 3 National Center for Neurological Disorders, Shanghai, China; 4 Department of Neurology, Xuanwu Hospital, Capital Medical University, Beijing, China; 5 Department of Neurology, Qingdao Municipal Hospital Group, Qingdao University, Qingdao, China

**Keywords:** Neurodegenerative diseases (NDDs), Early diagnosis, Blood biomarker, Deamidation, Isoaspartate, Human serum albumin (HSA)

## Abstract

**Background:**

The strongest risk factor of neurodegenerative diseases (NDDs) is aging. Spontaneous asparaginyl deamidation leading to formation of isoaspartate (isoAsp) has been correlated with protein aggregation in NDDs.

**Methods:**

Two cohorts consisting of 140 subjects were studied. Cohort 1 contained patients with AD and healthy controls, while Cohort 2 recruited subjects with mild cognitive impairment (MCI), vascular dementia (VaD), frontotemporal dementia (FTD), Parkinson’s disease (PD) and healthy controls. The levels of isoAsp in plasma human albumin (HSA), the most abundant protein in plasma, as well as the levels of immunoglobulin G (IgG) specific against deamidated HSA were measured. Apart from the memory tests, plasma biomarkers for NDDs reported in literature were also quantified, including amyloid beta (Aβ) peptides Aβ40 and Aβ42, neurofilament light protein (NfL), glial fibrillary acidic protein (GFAP) and phosphorylated tau 181 (p-tau181) protein.

**Results:**

Deamidation products of blood albumin were significantly elevated in vascular dementia and frontotemporal dementia (*P* < 0.05), but less so in PD. Intriguingly, the deamidation levels were significantly (*P* < 0.01) associated with the memory test scores for all tested subjects. Deamidation biomarkers performed superiorly (accuracy up to 92%) compared with blood biomarkers Aß42/Aß40, NfL, GFAP and p-tau181 in separating mild cognitive impairment from healthy controls.

**Conclusion:**

We demonstrated the diagnostic capacity of deamidation-related biomarkers in predicting NDDs at the early stage of disease, and the biomarker levels significantly correlated with cognitive decline, strongly supporting the role of deamidation in triggering neurodegeneration and early stages of disease development. Prospective longitudinal studies with a longer observation period and larger cohorts should provide a more detailed picture of the deamidation role in NDD progression.

**Supplementary Information:**

The online version contains supplementary material available at 10.1186/s40364-022-00435-8.

## Background

Deamidation of asparagine (Asn) residue is a spontaneous, non-enzymatic protein degradation process resulting in the formation of damaging isoaspartate (isoAsp). As isoAsp residues accumulate in proteins, the latter lose their native structure, function and solubility [1]. A body of research has demonstrated that the accumulation of isoAsp triggers protein aggregation, which ultimately leads to the onset and progress of multiple disorders, including neurodegenerative diseases (NDDs) [2–7]. One of the evidence was found in 1990s that the formation of isoAsp in amyloid beta (Aß) peptide, especially in position 7, facilitates its aggregation, and thus forming amyloid plaques in the brain [7].

NDDs, such as Alzheimer’s disease (AD), vascular dementia (VaD), frontotemporal dementia (FTD), and Parkinson’s disease (PD), are usually defined as the progressive loss of structure and function in neurons and axons of the central nervous system. The NDD diagnostics is usually based on clinical features, such as cognitive decline and movement disorders, as well as on neuroimaging techniques positron emission tomography (PET) and magnetic resonance imaging (MRI). Recently, molecular biomarkers reflecting protein pathology and aggregation has gained increased interest of NDD researchers. Apart from the cerebrospinal fluid (CSF) biomarkers (e.g., amyloid β (Aβ), tau, α-synuclein, etc.) obtaining of which is sometimes viewed as complicated, invasive and/or expensive, several blood biomarkers have emerged in recent years, such as neurofilament light protein (NfL) [8–10], phosphorylated tau (p-tau) [11–13] and glial fibrillary acidic protein (GFAP) [14–16]. However, the sensitivity and specificity of these blood biomarkers is not yet fully established, especially for early disease stages, and thus new reliable, specific, low-cost blood biomarkers are highly desirable. Besides, the majority of blood biomarker characterization studies concerned separating diagnosed AD from healthy controls, while much less results have been published on the more challenging task of diagnosing early neurodegeneration stages, such as mild cognitive impairment (MCI) ahead of diagnosed dementia.

Our recent findings have strengthened the link between isoAsp and AD pathology [17]. Using a novel enzyme-linked immunosorbent assay (ELISA) [18], we discovered that the accumulation of isoAsp in human serum albumin (HSA) leads to a diminished HSA capacity to carry Aß and p-tau, and thus to clear these damaging molecules in the blood circulatory system, which has been recognized as a major contributor to their accumulation in brain [19]. By studying an Amsterdam cohort, we have found that isoAsp levels in HSA strongly correlate with AD [17]. Furthermore, native immunoglobulins (IgGs) specific to deamidated HSA that could protect the human organism from the damaging effect of deamidation have been found significantly reduced in AD [17]. However, questions remained related to the performance of these biomarkers in non-Caucasian patients, specificity to AD of these biomarkers, and their ability to detect early disease stages.

To address these issues, we recruited a Shanghai cohort with several kinds of NDDs as well as subjects with MCI, and compared the performance of the deamidation biomarkers with other plasma NDD markers. The central hypothesis under verification was that accumulation of deamidation products due to breakdown of the isoAsp repair/removal mechanisms precedes most processes in AD initiation, and thus deamidation biomarkers might exhibit high sensitivity in detecting early stages of neurodegeneration.

### Study conclusions and implications

In this follow-up study we first confirmed on Shanghai cohort our earlier findings on Amsterdam cohort that isoAsp in plasma HSA is significantly increased (*P* < 0.0001) while the anti-aHSA IgG levels are lower (*P* < 0.0001) in AD patients compared to healthy controls. Then we measured the levels of these two biomarkers in other NDDs, such as VaD, FTD and PD, and found behavior similar to AD in the first two diseases (*P* < 0.01). However, in PD the results didn’t reach statistical significance, possibly due to a higher cognitive performance in PD group compared to other disease groups. Then we tested the performance of the deamidation biomarkers for MCI ahead of dementia diagnosis. This performance was found to be superior compared to other tested blood biomarkers, reaching the impressive accuracy of 92%. Then we tested the association of deamidation biomarkers with other analyzed parameters and discovered strong correlation with cognitive scores of the patients and controls. Taken together, these results strongly support the role of deamidation in NDD etiology, and open prospects of using deamidation biomarkers in early disease diagnostics.

## Methods

### Participants

Patients (*n* = 100: AD, 20; VaD, 20; FTD, 20; PD, 20; MCI, 20) in the study were recruited from the Huashan Hospital of Fudan University (Shanghai, China). Matched healthy controls (*n* = 40) were recruited from the Chinese Alzheimer’s Biomarker and LifestylE (CABLE; Qingdao, China) study previously described in detail [20–22]. The participants capable of communicating, cooperating with physical and cognitive examinations, and giving consent to the blood draw as well as other necessary ancillary diagnostic tests were included. We excluded participants with severe systemic illness or central nervous system diseases attributed to non-NDDs, such as central nervous system infectious diseases and epilepsy. All procedures conformed to the tenets set forth by the Helsinki Declaration. All participants or legal guardians gave their written informed consent. Ethics approval was received from the institutional review boards of each participating center.

### Diagnostic procedures

General cognitive tests including Mini-Mental Status Examination (MMSE), Montreal Cognitive Assessment (MoCA) were performed by neuropsychological professionals blinded to the study design and subsequent procedures. Participants without formal education completed the Montreal Cognitive Assessment-Basic (MoCA-B) questionnaire instead of the original MoCA. Some other tests critical for diagnosis like Clinical Dementia Rating scale (CDR), Activities of Daily Living (ADL), the Unified Parkinson’s Disease Rating Scale (UPDRS), and Frontal Behavioral Inventory (FBI) were also performed. Small portions of the translated international questionnaires were appropriately adjusted to ensure applicability to the Chinese Han population.

Diagnoses were made by experienced neurologists who were unaware of the research procedures. AD dementia was diagnosed according to the National Institute of Neurological and Communicative Disorders and Stroke and Alzheimer’s Disease and Related Disorders Association (NINCDS-ADRDA) criteria [23], as well as the National Institute on Aging-Alzheimer’s Association (NIA-AA) research framework [24]. All patients with AD dementia had strong PET evidence of the disease or CSF-confirmed Aβ-positive pathology. VaD was diagnosed according to the National Institute of Neurological Disorders and Stroke and the Association Internationale pour la Recherche et l’Enseignement en Neurosciences (NINDS-AIREN) criteria [25]. FTD was diagnosed according to the revised criteria set forth by the International Behavioral Variant of FTD Criteria Consortium [26]. PD was diagnosed according to the clinical criteria set forth by the International Movement Disorders Society (MDS) in 2015 [27]. MCI was diagnosed when there was objective evidence of cognitive decline while no significant evidence of impaired social or self-care ability. Healthy individuals recruited in the study were required to be matched to the patients with AD in terms of age and sex. In addition, all controls had an MMSE score > 24 at the screening visit and no signs of cognitive decline, thus not meeting the criteria for MCI or any NDD.

### Blood sample collection and analyses

All laboratory personnel were blinded to clinical information. To obtain plasma, venous blood samples collected in EDTA-containing tubes were centrifuged at 1800 rpm for 15 min at 4 °C. The supernatant was immediately removed, frozen in 200 μL aliquots, and stored at -80 °C until further processing.

Plasma Aβ42, Aβ40, p-tau181, GFAP, and NfL levels were quantified using single molecule arrays (SiMoA), an ultra-sensitive enzyme-linked immunosorbent assay (ELISA) technique on an automated SiMoA HD-X platform (Quanterix, Billerica, MA, USA). The SiMoA Human Neurology 4-Plex E assay was used to measure GFAP, Aβ40, Aβ42, and NfL levels, whereas p-tau181 was quantified by the SiMoA p-tau181 Advantage V2 assay.

### Quantification of isoAsp in blood HSA and IgGs against deamidated HSA

As described before [18], the plasma samples were sonicated, centrifuged and their protein concentrations were measured. All plasma samples and HSA standards were diluted to 5 μg/mL and analyzed in triplicates by an indirect Enzyme-Linked Immunosorbent Assay (ELISA). The isoAsp levels in each blood sample were calculated according to the standard curve of HSA constructed from standards on each plate. The results were normalized by the average value in the control group.

The IgG antibodies were purified from plasma using the Melon Gel IgG *P*urification Kit (Thermo Fisher Scientific, San Diego, CA, USA). To determine in each sample the amount of IgG against aged HSA (aHSA, ≈60% isoAsp), the purified IgG antibodies were used as the primary antibody in the indirect ELISA [17], while the secondary antibody was Goat anti-Human IgG (H + L) Secondary Antibody conjugated with HRP (Thermo Fisher Scientific, San Diego, CA, USA). To eliminate the effect of sample location on the plate, the data were normalized by the average reading in each row and column.

### Statistical analysis

The data analysis was performed using GraphPad Prism (version 8.0.2) and coding using R software (version 3.4.4). The parameters distributed normally were expressed as mean ± standard deviation (SD), and group differences were analyzed using two-tailed Student’s t-test. Otherwise, parameters were stated as medians with interquartile ranges, and a Mann-Whitney U test or a Kruskal-Wallis test was applied. The receiver operating characteristic (ROC) curves of Cohort 1 were adjusted for age, sex, education years and *APOE* ε4 carrier status according to the reported method [28, 29], while the ROC curves of Cohort 2 were adjusted for age, sex, and education years.

## Results

### Characteristics of participants

The baseline characteristics of the two cohorts acquired in the study are shown in Table 1. In Cohort 1, significant differences between the control and AD subjects were observed in cognitive scores MMSE and MoCA, as well as plasma biomarkers Aβ42, Aβ42/Aβ40, p-tau181, NfL and GFAP (*P* < 0.05), while no significant difference was found in plasma Aβ40. In Cohort 2, significant differences were discovered in MMSE, MoCA, plasma Aβ42, Aβ40, NfL and GFAP among the groups of control, MCI and other NDDs (*P* < 0.05), while no difference was observed among the groups in Aβ42/Aβ40 and p-tau181.

**Table 1:** Characteristics of participants in Cohort 1 and 2

Characteristic	Cohort 1		Cohort 2					
	Control (*n* = 20)	AD (***n*** = 20)	Control (*n* = 20)	MCI (*n* = 20)	VaD (*n* = 20)	FTD (*n* = 20)	PD (*n* = 20)	***P***
Age (years)	55.50 [54.00–60.25]	57.00 [53.75–59.00]	55.00 [52.75–63.00]	61.00 [56.00–70.25]	66.50 [59.50–72.25]*	60.50 [54.75–67.25]	67.50 [57.75–71.00]**	0.036
Women, n (%)	9 (45%)	12 (60%)	11 (55%)	13 [65%]	6 (30%)	13 (65%)	13 (65%)	0.113
Education years	9.00 [9.00–12.00]	9.000 [8.75–9.50]	9.00 [6.00–12.75]	9.00 [8.00–12.00]	9.00 [5.75–12.00]	9.00 [4.75–12.00]	9.00 [5.00–12.00]	0.687
*APOE* ε4 carriers, n (%)	1 (5%)	6 (30%)	2 (12%)	7 (44%)	6 (35%)	4 (24%)	NA	0.182
MMSE	28.50 [28.00–30.00]	14.50 [11.25–20.00]***	28.50 [27.00–30.00]	25.00 [24.00–27.25]**	17.00 [9.00–22.25]***	15.00 [9.00–18.00]***	23.00 [18.00–27.00]***	< 0.001
MoCA	26.00 [25.00–27.00]	7.50 [4.50–13.00]***	26.00 [24.75–27.00]	20.50 [17.75–23.00]***	9.50 [5.00–13.25]***	7.00 [4.00–10.00]***	16.00 [9.000–20.00]***	< 0.001
plasma Aβ42 (pg/mL)	4.60 [4.28–6.14]	4.20 [3.05–4.96]*	4.13 [3.33–5.55]	4.73 [3.67–6.54]	4.90 [4.16–6.27]	6.19 [4.75–7.17]*	6.72 [5.63–7.43]**	0.008
plasma Aβ40 (pg/mL)	83.35 [70.67–100.19]	88.24 [75.36–111.53]	80.34 [68.35–91.46]	86.79 [74.37–113.72]	83.97 [75.11–104.68]	101.66 [87.08–112.10]*	111.18 [95.56–124.03]**	0.011
plasma Aβ42/Aβ40 (pg/mL)	0.06 [0.05–0.07]	0.04 [0.04–0.05]***	0.06 [0.05–0.07]	0.05 [0.04–0.06]	0.06 [0.05–0.06]	0.06 [0.05–0.07]	0.06 [0.05–0.07]	0.349
plasma p-tau181 (pg/mL)	1.80 [1.33–2.21]	3.81 [3.20–5.54]***	2.14 [1.94–2.84]	1.68 [1.36–2.16]*	1.71 [1.14–2.29]*	1.76 [1.55–2.08]*	1.53 [1.28–2.24]*	0.079
plasma NfL (pg/mL)	10.76 [8.79–12.66]	24.81 [15.46–30.93]***	12.53 [9.94–19.92]	17.72 [11.24–21.02]	43.07 [31.77–89.99]***	51.05 [31.54–84.92]***	26.09 [17.40–34.60]**	< 0.001
plasma GFAP (pg/mL)	73.04 [51.99–92.33]	266.84 [182.27–318.94]***	64.33 [45.11–101.08]	113.87 [78.58–132.65]**	119.97 [85.83–156.38]**	144.54 [99.90–183.39]***	124.48 [105.50–175.45]***	< 0.001

*P < 0.05; **P < 0.01; ***P < 0.001 (compared to controls)

### Validating the efficacy of deamidation biomarkers in AD diagnostics

**Fig. 1:**
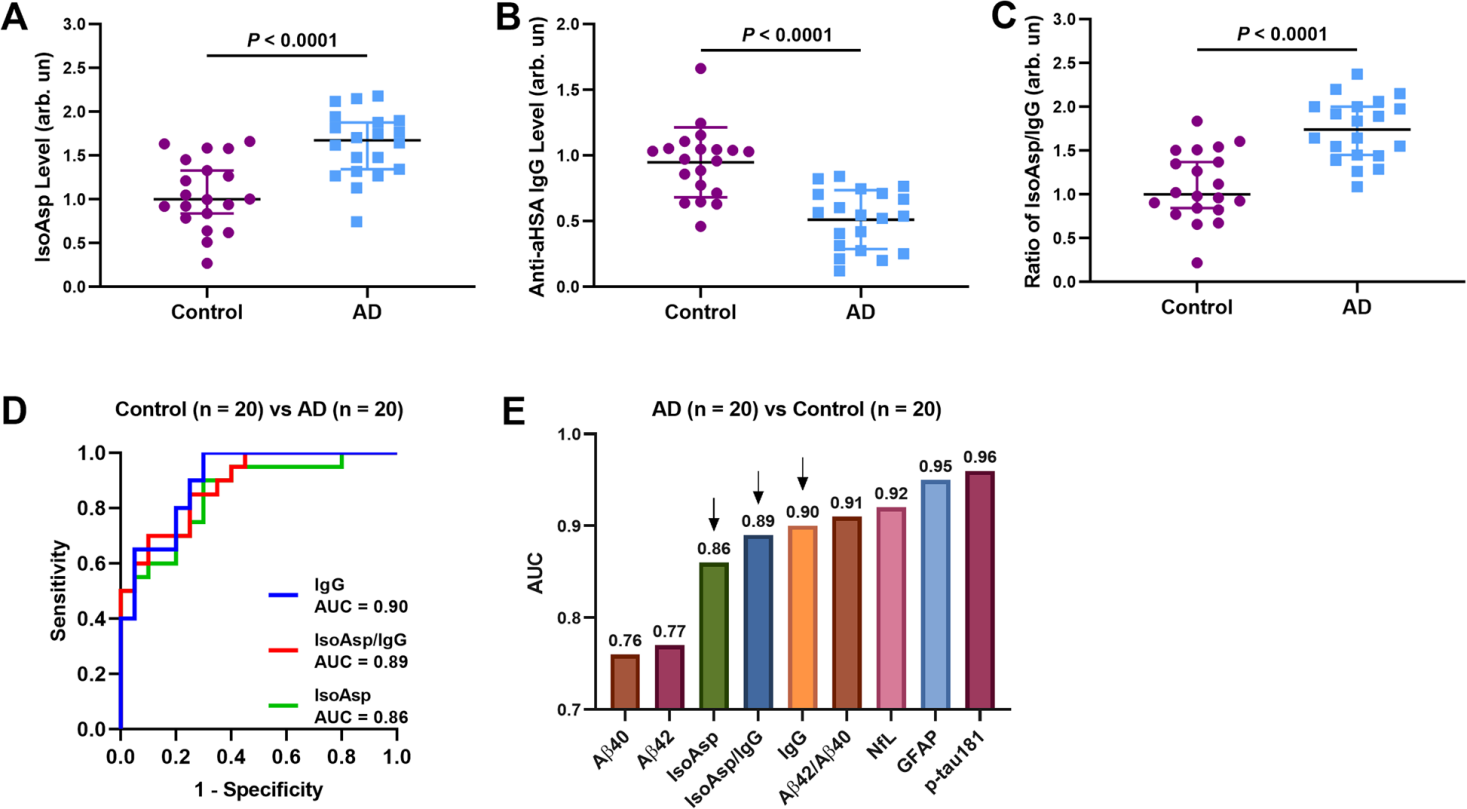
Validating the deamidation biomarkers in differentiating AD vs Control. **a-c** IsoAsp levels in HSA are increased (**a**), and the anti-aHSA IgG levels are decreased in AD compared to Control (**b**); the isoAsp/anti-aHSA IgG ratios are also different (**c**). The ROC curves for differentiating AD and controls by (**d**) isoAsp in HSA, anti-aHSA IgG and isoAsp/anti-aHSA IgG ratio. (**e**) The comparison of AUCs of all blood biomarkers

Cohort 1 data showed the increased levels of isoAsp in HSA (Fig. 1a) and the deficiency of IgGs against deamidated HSA in AD blood compared to controls (Fig. 1b), validating the results from Amsterdam cohort [17]. The normalized signals reflecting the isoAsp level in plasma HSA were increased by 9% on average (*P* < 0.0001) while the average signal of anti-aHSA IgG was lower by 6% (*P* < 0.0001) in AD patients compared to healthy donors. In parallel, the isoAsp/anti-aHSA IgG (IsoAsp/IgG) ratio was 15% higher in AD than controls (*P* < 0.0001, Fig. 1c). The diagnostic values of the three above parameters were assessed using ROC curves and the area under the curve (AUC) values (Fig. 1d), with AUC interpreted as the diagnostic accuracy. All of the AUCs were above 0.80, suggesting a good differentiation between AD and control. We also compared the AUCs of deamidation parameters with other plasma biomarkers, and the former significantly surpassed Aß40 and Aß42, but somewhat underperformed NfL, GFAP and p-tau181, the AUCs of which were above 0.9 (Fig. 1e).

### Diagnostic value of deamidation biomarkers for other NDDs

**Fig. 2:**
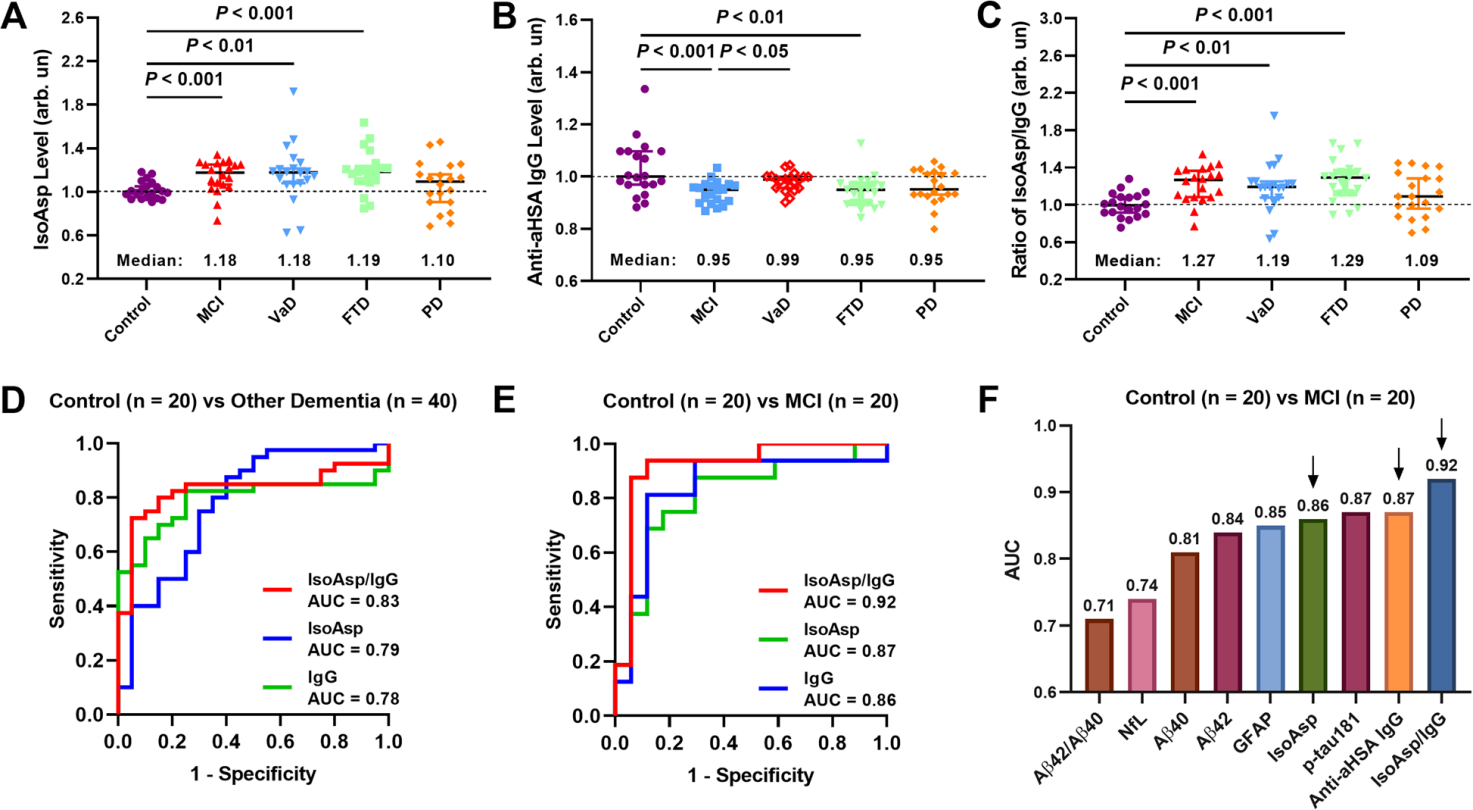
Differentiation among various NDDs and disease stages. **a-c** Distinguishing Control from MCI and Other NDDs (VaD, FTD and PD). **d-f** The ROC curves for differentiation between (**d**) Other dementia (VaD and FTD) and control, (**e**) MCI and control. **f** The comparison of AUCs of all blood biomarkers

In comparison between control in Cohort 2, the isoAsp levels in plasma HSA were significantly elevated (by ≥18%) in MCI (*P* < 0.001), VaD (*P* < 0.01) and FTD (*P* < 0.001), but not in PD despite a 10% average elevation (Fig. 2a). The anti-aHSA IgG levels were found to be significantly lower in MCI (*P* < 0.001) and FTD (*P* < 0.01), but not in VaD and PD (Fig. 2b). The IsoAsp/IgG ratios also showed a significant difference in MCI (*P* < 0.001), VaD (*P* < 0.01) and FTD (*P* < 0.001), with median values ≥19% compared to control (Fig. 2c). No significant difference was observed for PD despite an elevated by 9% average value, possibly due to the larger spread of data within PD group and the higher memory test scores compared with other groups of patients.

The AUCs of ROC curves of different comparisons were calculated (Fig. 2d-g). Collectively, the deamidation biomarkers showed reasonable efficacy (AUC = 0.78..0.83) in diagnosing other dementia types than AD (Fig. 2e).

### Diagnostic values of deamidation biomarkers for MCI

A very promising efficacy (AUC = 0.86..0.92) was obtained by deamidation biomarkers for separating MCI ahead of dementia diagnosis from healthy controls (Fig. 2d). More specifically, the IsoAsp/IgG ratio ranked the first among all plasma biomarkers (AUC = 0.92), followed by anti-aHSA IgG (AUC = 0.90), p-tau181 (both AUC = 0.87) and isoAsp (AUC = 0.86) (Fig. 2f). For comparison, all AUCs of the plasma biomarkers for early Aß pathology studied in a recent work (p-tau181, p-tau231, p-tau217, NfL, GFAP and Aß42/Aß40) were below 0.86 [30].

### Association of deamidation biomarkers with other cognitive parameters

**Fig. 3:**
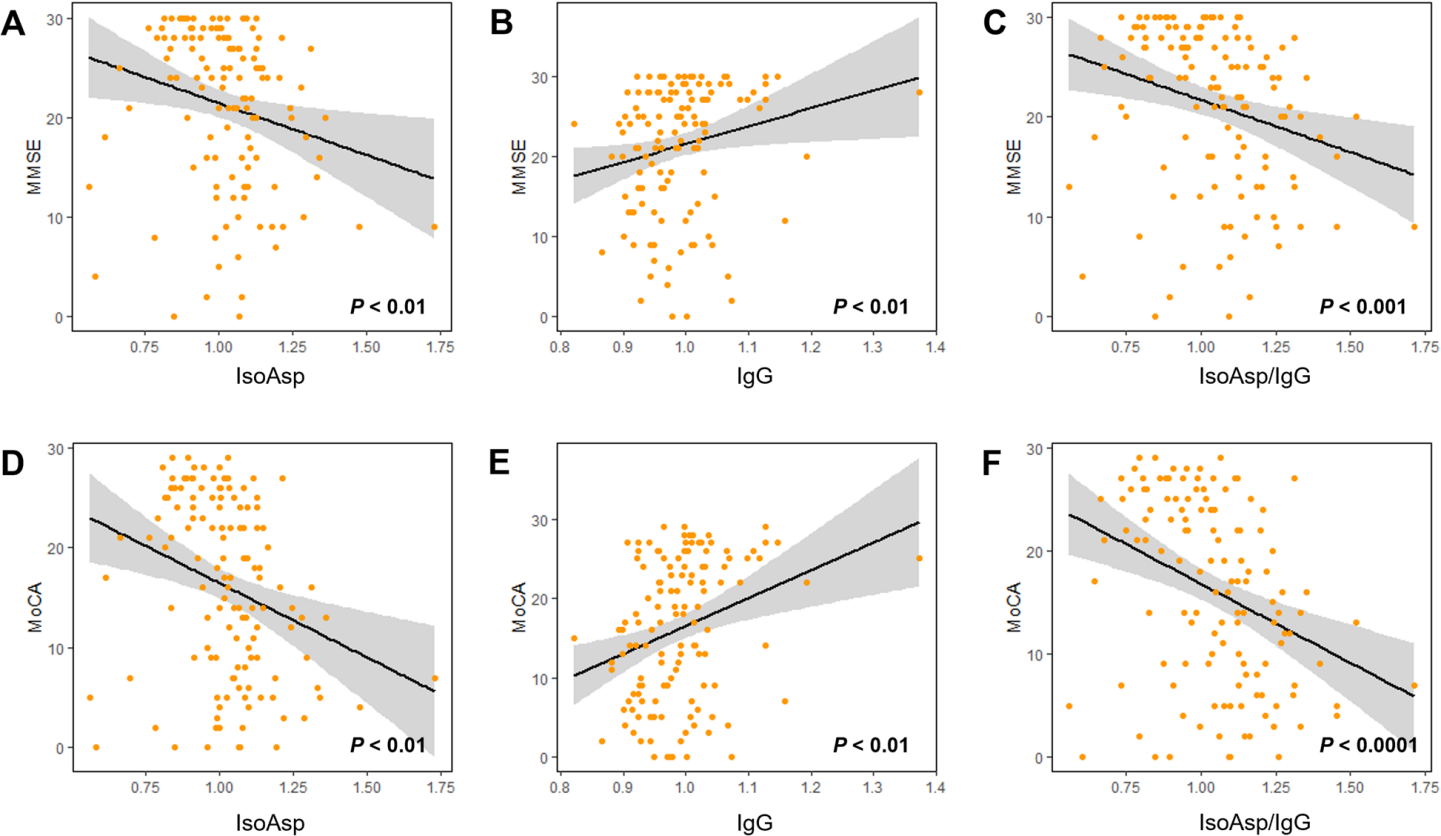
Association deamidation biomarkers with memory scores. Linear regression analyses for MMSE score (**a-c**) and MoCA score (**d-f**) versus (**a**, **d**) isoAsp in HSA, (**b**, **e**) anti-aHSA IgG, (**c**, **f**) isoAsp/anti-aHSA IgG ratio

Strong positive correlations were discovered in both cohorts between cognitive scores (MMSE and MoCA) and the anti-aHSA IgG levels (*P* < 0.01), while there was a significant negative correlation between cognitive scores and the isoAsp levels in plasma HSA as well as IsoAsp/IgG ratio (*P* < 0.01), supporting the link between deamidation and the early onset of disease pathology (Fig. 3a-f). We also found a negative relationship of anti-aHSA IgG with plasma Aß40, Aß42/Aß40 ratio, GFAP and NfL, while both isoAsp and the IsoAsp/IgG ratio were positively associated with them (Supplementary Table S1).

## Discussion

The findings in this work validated the previous discovery of the elevated isoAsp levels in HSA and reduced anti-aHSA IgG in AD plasma compared to controls [17]. Furthermore, we explored the potential of deamidation biomarkers in other NDDs and in early diagnostics of dementia. The isoAsp levels were found significantly elevated in MCI, FTD and VaD (*P* < 0.01) and the anti-aHSA IgG levels were reduced in MCI and VaD (*P* < 0.01). The common causative link between these different types of dementia seems to be the failure of isoAsp clearance. A possible exception was PD, in which the deamidation-related biomarkers changed moderately without reaching statistical significance. This could be due to the fact that PD has distinctly different disease trajectory from other dementia types [27].

Arguably, the most significant finding was the superior performance of the deamidation indicators among all tested blood biomarkers in detection of MCI ahead of dementia diagnosis. However, the cohort size here was small, thereby requiring validation of the results with a larger cohort in the future. Nevertheless, together with a strong relationship between the deamidation biomarkers and cognitive parameters in all 140 subjects, this finding supports the role of deamidation in dementia initiation.

Indeed, pathological protein aggregation and abnormal accumulation intra- or extracellularly are the common characteristics shared in NDDs at autopsy. These protein aggregates participate in progressive neuronal dysfunction, neuroinflammation and programmed cell death, occurring before the initiation of clinical features [31, 32]. The disease-specific proteins can be natively unfolded (e.g., microtubule tau protein in AD and FTD) or formed via partially unfolded intermediates (Aß in AD). In all cases deamidation and isoaspartate formation can enhance the aggregation rate as the polypeptide backbone extension resulting from the methylene rearrangement from the side chain disrupts the protein structure. As HSA is the most abundant protein in blood and participates in clearance of molecules implicated in the NDD onset, such as Aß and p-tau [17, 33], HSA aggregation caused by deamidation and the resulting loss of clearance capacity may be one of the main risk factors for NDDs.

Moreover, deamidation is one of the causes of human autoimmune response since isoaspartate is immunogenic [34, 35], which may cause T cells circumvent the non-self/self-barrier that eventually cause age-related autoimmune diseases [36]. For example, deamidation of myelin proteins has been found to initiate the immune response leading to multiple sclerosis [37]. Therefore, it will be of interest to investigate the role of deamidated HSA in autoimmune diseases in the future.

**Fig. 4:**
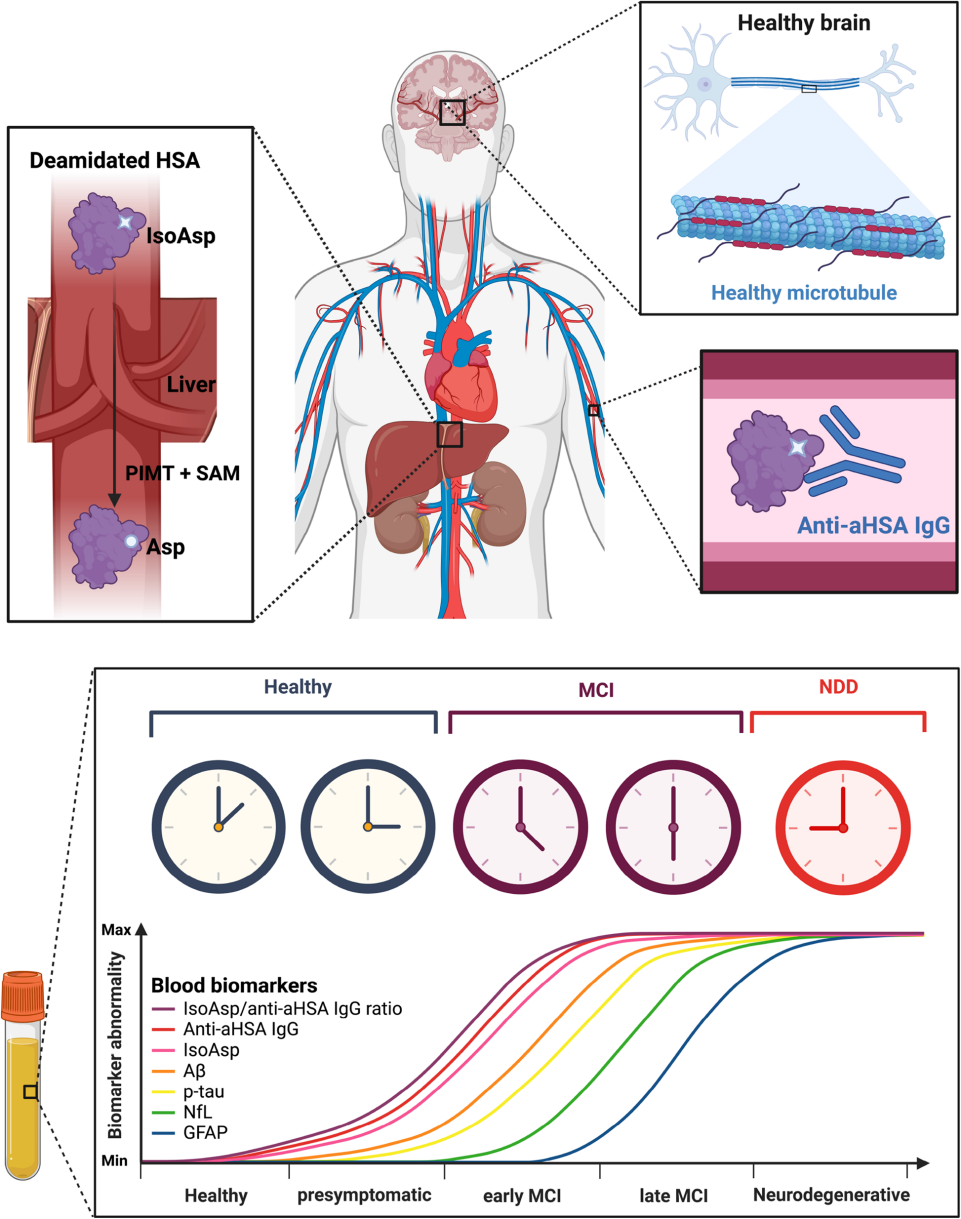
The model connecting the overall state of isoAsp repair/removal in proteins with the onset of neurodegenerative diseases, supporting early disease diagnostics by deamidation biomarkers. (Created with BioRender.com)

Summarizing our previous work and current findings, we propose the following deamidation-driven mechanism of NDD initiation (Fig. 4). In a healthy body, spontaneously deamidated HSA is repaired by the enzyme PIMT in liver using SAM as a cofactor; the anti-aHSA antibodies in blood remove deamidated HSA, and the newly synthesized HSA molecules restore homeostasis. When in aging the SAM levels become insufficient due to reduced synthesis and increased consumption [38], PIMT-mediated isoAsp repair diminishes, the isoAsp starts to accumulate in blood proteins, depleting the stock of the native antibodies against isoAsp. This causes reduced removal of deamidated HSA and formation of HSA aggregates [17]. Deamidated and aggregating HSA has a diminished capacity of binding with Aβ, p-tau, metal ions and other molecules both in blood and CSF, reducing their clearance from brain and contributing to their enhanced aggregation there [17]. It is also plausible that the reduced isoAsp repair and removal of deamidation products concerns brain proteins involved in maintaining cognitive functions. Therefore, the isoAsp level in blood may act as a “thermometer” for the state of protein health in general, reflecting both cognitive decline and the onset of neurodegenerative diseases. Importantly, such “thermometer” may start showing abnormal values long before the onset of clinical symptoms and monitor the cognitive decline, thus probably enabling early detection of slight cognitive impairment via a non-invasive and scalable tool, which has been called for recently [39].

## Conclusion

This study validated our previous findings of the important role of blood protein deamidation in AD and discovered such a role in other NDDs, possibly excluding PD. An important finding is the promising performance of the deamidation biomarkers, namely levels of isoAsp and anti-aHSA IgGs, as well as the IsoAsp/IgG ratio, in early stages of mental decline and a significant correlation with mental scores. The cohort size in this study was relatively small. Thus, it is worth to further investigate the potential of these biomarkers in early NDD diagnostics in a broader longitudinal study capable of depicting a more nuanced picture of deamidation role in disease progression. Furthermore, in a future study more MCI participants should be recruited with follow-up data available to investigate the ability of deamidation biomarkers to predict the progression from MCI to probable AD. More types of NDD should be investigated, such as Huntington’s disease, multiple sclerosis, amyotrophic lateral sclerosis, spinocerebellar ataxias, etc., to fully define the specificity of deamidation biomarkers in NDD diagnostics.

## Supplementary Information

**Additional file 1:** **Table S1.** The regression coefficients (R) between deamidation biomarkers and other indicators.

## Data Availability

All data generated or analyzed during this study are included in this publication and/or are available from the corresponding author on reasonable request.

## Abbreviations

*Aβ*: Amyloid beta
*AD*: Alzheimer’s disease
*aHSA*: Aged human serum albumin
*APOE ε4*: Apolipoprotein ε4
*Asn*: Asparagine
*Asp*: Aspartate
*AUC*: Area under curve
*ELISA*: Enzyme-linked immunosorbent assay
*FTD*: Frontotemporal dementia
*GFAP*: Glial fibrillary acidic protein
*HSA*: Human serum albumin
*IgG*: Immunoglobulin G
*IsoAsp*: Isoaspartate
*IsoAsp/IgG ratio*: Isoaspartate/Anti-aged-human-serum-albumin immunoglobulin G ratio
*MCI*: Mild cognitive impairment
*MMSE*: Mini-Mental Status Examination
*MoCA*: Montreal Cognitive Assessment
*NDDs*: Neurodegenerative diseases
*NfL*: Neurofilament light protein
*PD*: Parkinson’s disease
*PIMT*: Protein isoaspartyl methyltransferase
*p-tau*: Phosphorylated tau
*ROC*: Receiver operating characteristic
*SAM*: S-adenosylmethionine
*SD*: Standard deviation
*VaD*: Vascular dementia
