# Supplementary material for: Deamidation-related blood biomarkers show promise for early diagnostics of neurodegeneration
Source: Biomark Res. 2022 Dec 27;10:91. doi: 10.1186/s40364-022-00435-8 (PMC9795668; doi:10.1186/s40364-022-00435-8)
Supplement: Supplementary file 1 — Additional file 1: Table S1. The regression coefficients (R) between deamidation biomarkers and other indicators. [file 40364_2022_435_MOESM1_ESM.docx]

**Supplementary Table S1. The regression coefficients (R) between deamidation** **biomarkers and other indicators.**


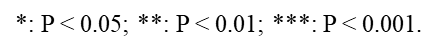

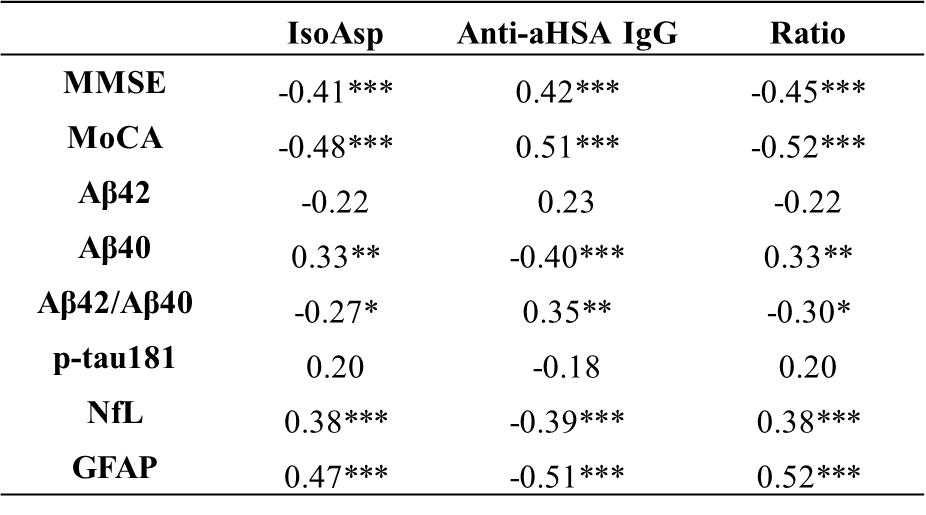


Abbreviation: MMSE, Mini-Mental Status Examination; MoCA, Montreal Cognitive Assessment; aHSA, aged human serum albumin; IsoAsp, isoaspartate; IgG, immunoglobulin G; Aβ, amyloid beta; p-tau, phosphorylated tau; NfL, neurofilament light protein; GFAP, glial fibrillary acidic protein; Ratio, the IsoAsp/Anti-aHSA IgG ratio.
